# Dataset on FAEE synthesized from oil blends via a derived submerged fermented *Theobroma cacao* pod husk: Application of hybrid design optimizer

**DOI:** 10.1016/j.dib.2022.108349

**Published:** 2022-06-09

**Authors:** Adepoju T. F, Ukpong A. A, Balogun T. A, Emberru E. R

**Affiliations:** a Chemical Engineering Department, Federal University, Otuoke, Bayelsa State, Nigeria, P.M.B 126, Yenagoa, Nigeria; b Chemical/Petrochemical Engineering Department, Akwa-Ibom State University, Ikot Akpaden, Mkpat Enin L.G.A., Akwa-Ibom State. Nigeria. P.M.B 1167, Uyo, Nigeria; c Chemical Engineering Department, River State University, Port Harcourt, River State, Nigeria

**Keywords:** Blends, Fatty acid ehthyl ester, Catalyst, Transformation, Mathematical optimization, Hybrid design, *Theobroma cacao* pod husk

## Abstract

A new base catalyst was derived from submerged fermented *Theobroma cacao* pod husk (TCPH) in this work, the developed catalyst was tested for the production of fatty acid ethyl ester (FAEE) using the blends of beef tallow (BTO) - waste use oil (WUO) in the ratios of 10:90 (BTO_10_)_,_ 20:80 (BTO_20_)_,_ 30:70 (BTO_30_)….., 90:10 (BTO_90_)_,_ respectively. To determine the optimum FAEE yield with variable effects (reaction time, catalyst amount, reaction temperature, and EtOH/OMR), a hybrid design from response surface was adopted with coefficient of determination (R^2^) and Adjusted coefficient of determination (R^2^-adj.). Furthermore, the catalytic efficiency was tested by catalyst recycle, refining, and reusability test.

Data revealed the oil blend ratio of BTO_60_:WUO_40_ was adequate to produced low viscous FAEE. Catalysts' analysis and characterization revealed the catalyst produced high CaO-base of 87.65 (%wt.), which accounted for the high yield of FAEE. Mathematical optimization via hybrid design showed that the catalyst amount with high F-value = 3063.24 and P-value = 0.0115, played the most significant role in the conversion of blended oil to FAEE among the variable factors considered. Furthermore, based on Box-Cox transformation, the lambda indicated a normal data in normal function of Y^3^ for polynomial model accuracy. Optimum validated FAEE yields of 99.64 (%wt.) with high coefficient of determination (R^2^) was established. The qualities of the FAEE were within the standard specification for biodiesel, and the produced catalysts can serve as feedstocks for industrial application.

## Specifications Table

**Table:** 

Subject	Energy
Specific subject area	Renewable and Sustainable Energy
Type of data	Table, Figure
How data were acquired	The blend of oil was obtained via ratio blending 10:90 (BTO_10_)_,_ 20:80 (BTO_20_)_,_ 30:70 (BTO_30_)….., 90:10 (BTO_90_)_,_ respectively. Developed catalyst was synthesized from calcined *Theobroma cacao* pod husk. Catalyst analysis and characterization were carried out using SEM, FTIR, XRD, and BET analysis. The design of experiment, effects of variables, determination of coefficient of determination and process variable optimization to evaluate the optimum FAEE was obtained using hybrid design. Analysis of variance (ANOVA) was used to confirmed variable significant. Quality of FAEE was determined through AOAC (1997) [1] standard methods. The efficiency of the catalyst was carried out through refining, purification and reusability test. The quality of FAEE was ascertained by comparing with biodiesel recommended standard [2], [3].
Data format	Raw, Analysed
Description of data collection	WUVO was made clean by heating and filtration. Beef tallow was made into pure beef tallow oil. Cocoa husk was calcined. Oil blend was carried the ratio in the ratio of 10:90_,_ 20:80…..90:10 [5]. The synthesis of FAEE was via transesterification method [4]. Catalyst basic strength was tested by reusability test. The qualities of FFA meet biodiesel standard [2], [3]. All raw data are within the article.
Data source location	Chemical Engineering Department Federal University Otuoke, Bayelsa State, Nigeria, P.M.B 126, Yenagoa, Nigeria.
Data accessibility	With the article

## Value of the Data

• Data on mixed ratio serve as a guide for synergy required to blend oil for industrial processes.
• Dataset acquired from the derived bio-base from calcined fermented *Theobroma cacao* pod husk be used catalytic material in petroleum industries for biofuel conversion.
• Dataset on biodiesel can help to model and optimized the process variable for optimum FAEE yield and the interactions among the variables.
• Dataset obtained from the use of *Theobroma cacao* pod husk shows that fermentation increase the percentage of bio-base derived from the husk.
• Dataset on quality characterization of FAEE shows that the produced FAEE can serve as replacement for conventional diesel.

## 1 Data Description

This article produce dataset on different mixed ratio of waste oil with fat oil carried out at the interval of ten step increment (Table 1). The table also produced data on the physicochemical properties of the oil so as to determine the mixed oil with less viscosity, low acid value and moderate density. Table 2 produced data on experimental design with different variables level and factor as generated by hybrid design (expert 6.0.8 trial version). Four factors-five level was considered and these generate 16 experimental runs data with FAEE as response variable data. Table 3a show the dataset on the experimental and predicted yield generated by the design expert used for process optimization for test of significant, while Table 3b reflect the dataset on the analysis of variance (ANOVA) for every variable test of significant with p value< 0.005. Datasets on coefficient of determination (R-Squared), adjusted coefficient of determination (Adj. R-Squared), predicted coefficient of determination (Pred. R-Squared), and the adequate precision (Adeq. Precision) used for variable interactions an how mutaual the interaction of one is superir to the others are also presented in the table. Meanwhile, the second-order mathematical differential equation that correlated the response variable (FAEE3) data with the constraint variables (X_1_: reaction time, X_2_: catalyst amount, X_3_: reaction temperature, and X_4_: EtOH/OMR) are presented in Eqn. (1).

**Table 1:** Data of physicochemical properties of the oil blend.

Blends	Physicochemical Properties						
Ratio (BTO: WUVO)	MC (%)	SG	V @ 40 °C (mm^2^/s)	AV (mgKOH/g oil)	SV (mg KOH/g oil)	IV (meq O_2_/kg oil)	API g
BTO_10_	0.020	0.914	25.80	0.332	192	60.18	23.31
BTO_20_	0.020	0.912	24.90	0.303	189	60.03	23.65
BTO_30_	0.020	0.910	24.60	0.296	185	59.86	23.99
BTO_40_	0.020	0.905	23.50	0.283	186	59.80	24.85
BTO_50_	0.020	0.904	23.10	0.262	185	59.56	25.03
***BTO_60_***	***0.020***	***0.890***	***22.30***	***0.249***	***180***	***58.88***	***27.49***
BTO_70_	0.020	0.902	22.52	0.252	186	58.60	25.37
BTO_80_	0.020	0.911	22.86	0.272	188	59.94	23.82
BTO_90_	0.020	0.913	22.94	0.276	191	59.70	23.48

M = Moisture content, SG = Specific gravity, V = Viscosity, AV = Acid value, IV = Iodine value, PV = Peroxide value, SV = Saponification value, API g = API gravity.

**Table 2:** Five level- four variable- factors experimental for FAEE.

					Levels			
Variables	Units	Symbol	-2	-1		0	1	2
Reaction time	(min)	$X_{1}$	60	65		70	75	80
Catalyst amount	(wt.%)	$X_{2}$	1.5	2.0		2.5	3.0	3.5
Reaction temp.	(°C)	$X_{3}$	60	65		70	75	80
EtOH/OMR	(ml/ml)	$X_{4}$	4	5		6	7	8

**Table 3a:** Experimental data and the predicted value.

SN	X_1_	X_2_	X_3_	X_4_	FAEE3	PFAEE3
1	0.000	0.000	0.000	1.732	92.30	92.30
2	0.000	0.000	0.000	-0.269	93.80	93.80
3	-1.000	-1.000	-1.000	0.604	90.50	90.46
4	1.000	-1.000	-1.000	0.604	93.00	93.04
5	-1.000	1.000	-1.000	0.604	91.60	91.64
6	1.000	1.000	-1.000	0.604	91.70	91.66
7	-1.000	-1.000	1.000	0.604	90.90	90.94
8	1.000	-1.000	1.000	0.604	96.24	96.20
9	-1.000	1.000	1.000	0.604	97.20	97.16
**10**	1.000	1.000	1.000	0.604	**99.80**	**99.84**
11	1.518	0.000	0.000	-1.050	99.40	99.40
12	-1.518	0.000	0.000	-1.050	93.00	93.00
13	0.000	1.518	0.000	-1.050	96.00	96.00
14	0.000	-1.518	0.000	-1.050	86.80	86.80
15	0.000	0.000	1.518	-1.050	97.90	97.90
16	0.000	0.000	-1.518	-1.050	85.00	85.00

**Table 3b:** Anova and test of significant table.

Source	Sum of Squares	df	Mean Square	F -Value	Prob > F
Model	263.03	14	18.79	1300.18	0.0217
$X_{1}$	32.54	1	32.54	2251.71	0.0134
$X_{2}$	44.26	1	44.26	3063.24	0.0115
$X_{3}$	108.11	1	108.11	7481.75	0.0074
$X_{4}$	0.56	1	0.56	38.91	0.1012
$X_{1}^{2}$	12.09	1	12.09	836.54	0.0220
$X_{2}^{2}$	2.80	1	2.80	193.46	0.0457
$X_{3}^{2}$	2.62	1	2.62	181.25	0.0472
$X_{4}^{2}$	2.20	1	2.20	152.05	0.0515
$X_{1}X_{2}$	3.30	1	3.30	228.54	0.0420
$X_{1}X_{3}$	3.56	1	3.56	246.67	0.0405
$X_{1}X_{4}$	1.83	1	1.83	126.57	0.0564
$X_{2}X_{3}$	12.65	1	12.65	875.46	0.0215
$X_{2}X_{4}$	9.72	1	9.72	672.64	0.0245
$X_{3}X_{4}$	12.68	1	12.68	877.26	0.0215
Residual	0.014	1	0.014	-	-
Cor Total	263.04	15	-	-	-

	Fit Statistics				

Std. Dev.	0.12		R-Squared		99.98%
Mean	93.45		Adj R-Squared		99.92%
C.V.	0.0056		Pred R-Squared		99.97%
PRESS	0.0043		Adeq Precision		127.523

**Fig. 1:**
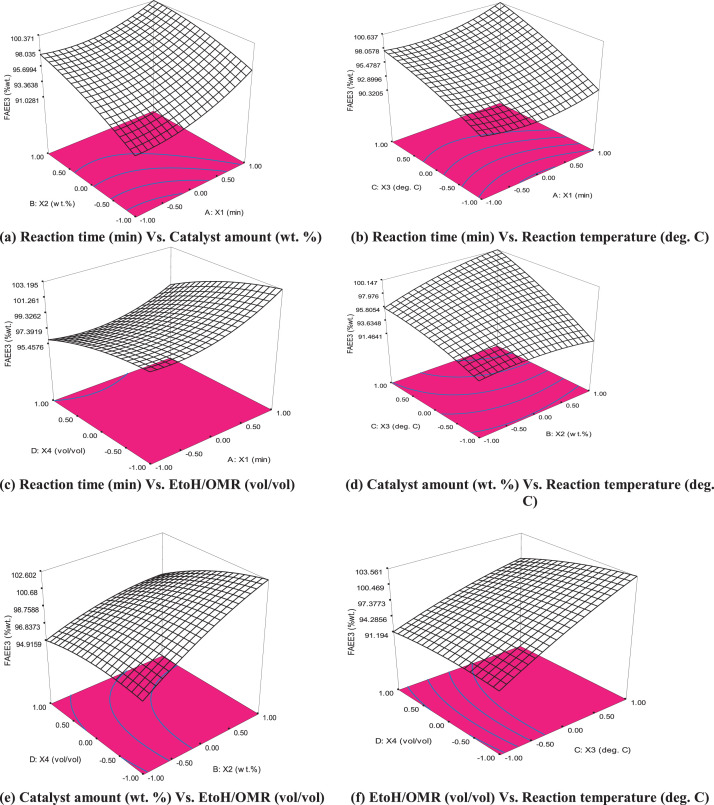
(a-f): Three dimensional plots.

**Fig. 2:**
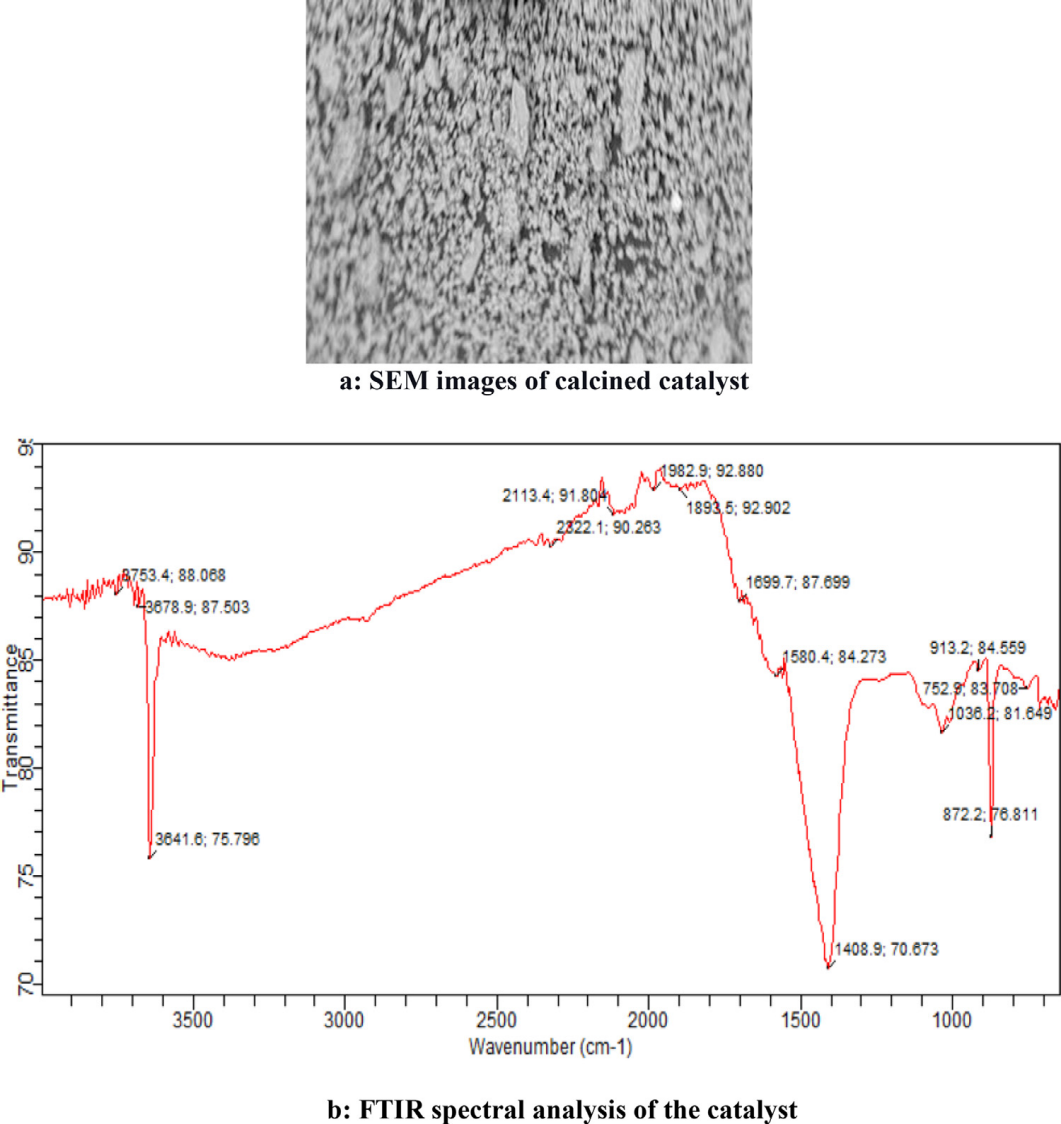
a: SEM images of calcined catalyst. b: FTIR spectral analysis of the catalyst.

**Fig. 3:**
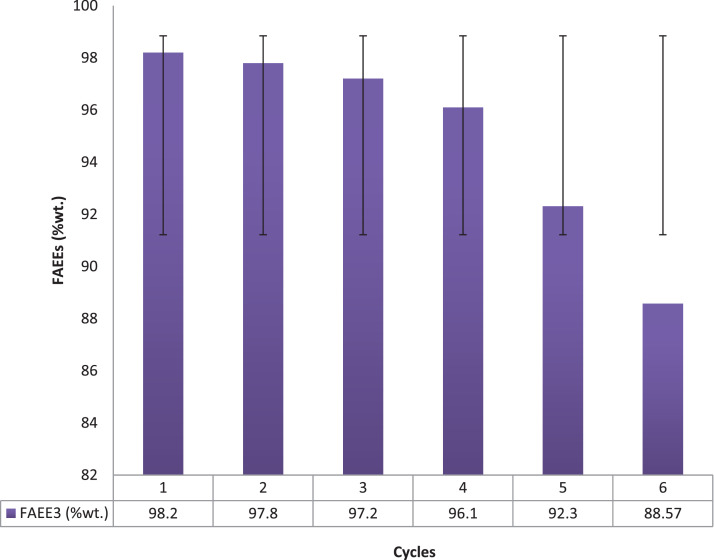
Plots of data of catalysts reusability test.

The graphical interactions between the responses (FAEE3) data and the linear constraint interactions ($X_{1}X_{2}$, $X_{1}X_{3\;}$, $X_{1}X_{4}$, $X_{2}X_{3}$, $X_{2}X_{4}$, and $X_{3}X_{4}$) dataset known as three-dimensional contour plots are presented in Fig. 1(a-f). Data on morphological characteristic analysis of derived bio base catalyst used for FAEE synthesis via SEM are presented in Fig. 2(a), while the data functional groups that verify the presence of absorption bands spectrum of base catalyst via FTIR analysis are presented in Fig. 2b. Dataset on Table 4 shows the BET analysis data on the surface, porous volume, basicity and the percentage composition of base present in the derived catalyst obtained via nitrogen adsorption-CO_2_ TPD. Data plot on catalyst recycle, refining, reusability test are plotted and displayed in Fig 3, which showed the strength of catalyst renewability. Table 5 however described the dataset obtained from oil mixed properties, FAEE qualities as compared with recommended biodiesel standard (ASTM D6751 and EN 14214). Based on dataset on comparative study of this work with other research earlier reported data, Table 6 indicate vividly the superiority of datasets obtained in this study as compare with other reports.

(1) $$\begin{array}{ccc} \text{FAEE}3\left(\%\text{wt}.\right) & = & +\;93.90+1.61X_{1}+1.87X_{2}+2.93X_{3}-0.21X_{4}-0.64X_{1}X_{2}+0.67X_{1}X_{3\;}\\ & & -\;0.48X_{1}X_{4}+1.26X_{2}X_{3}-1.10X_{2}X_{4}-1.26X_{3}X_{4}+1.41X_{1}^{2}-0.68X_{2}^{2}\\ & & -\;0.65X_{3}^{2}-0.66X_{4}^{2} \end{array}$$

**Table 4:** BET and XRD analysis of the catalyst.

Catalysts	β (m^2^/g)	λ (cm^3^/g)	CaO (%)	BS (μmole.g^−1^) 400<BS<650 >650		TBS	BSD (μmole/m^2^)	FAEE (%wt.)	CA (wt.%)
SFCTCPH	1.10	0.0030	87.65	22	174	196	178.18	98.20	2.50

**β** = Surface area, **λ** = Pore volume, BS = Basic site, TBS = Total basic site, BSD = Basic site density, GD = Green diesel, CA = Catalyst amount.

**Table 5:** Qualities of the produced FAEE.

Parameter	BTO60	FAEE3	[2]	[3]
Colour@ 27 oC	Brownish-yellow	Light yellowish	-	-
State @ room temp	Liquid	Liquid	Liquid	Liquid
Specific gravity	0.902	0.864	-	860-900
Viscosity @ 40 ^o^C/ (mm^2^/s)	22.30	2.78	1.9-6.0	3.5-5.0
Moisture content (%)	0.02	<0.01	<0.03	0.02
%FFA (as oleic acid)	0.1745	0.018	0.40 max	0.25 max
Acid value (mg KOH/g oil)	0.249	0.036	0.80 max	0.50 max
Iodine value (g I_2_/100g oil)	58.88	53.62	ND	120 max
Saponification value (mg KOH/g oil)	180.00	172.22	236.66-253.04	ND
Peroxide value (meq O_2_/kg oil)	12.65	8.60	ND	12.85
HHV (MJ/kg)	41.17	41.52	ND	ND
Cetane number	63.39	65.92	57 min	51 min
API gravity	22.30	32.27	30-42	ND
Diesel index	49.50	52.04	50.4 min	ND

ND = Not Determine.

## 2 Experimental Design, Materials and Methods

For oil mix ratio, a blend ratio of step length of ten was adopted 10:90; 20:80; 30:70 …….90:10 of waste used oil to beef tallow oil was used to obtain a low viscous oil, low acid value and low density required for FAEE synthesis. For the experimental design, a hybrid design under response surface methodology (expert 6.0.8 trial version) was employed to design the experiment with consideration of four factors namely; reaction time, catalyst amount, reaction temperature and ethanol to oil molar ratio, respectively, was used to study the effects of variables on the response (FAEE) in a single batch reaction.

Materials used in this work are Ethanol, Methanol, Hydrochloric acid, Sulphuric acid, Sodium thiosulphate, Sodium hydroxide, Starch solution, Wij's solution, and phenolphthalein etc., obtained from ChemiSciences Nig. Ltd.  Further material also include waste used oil (WUVO), beef tallow oil (BTO), and Cocoa pod husk (*Theobroma cacao* pod husk).

Equipment adopted are three necked batch reactor for transesterification of oil to FAEE, scanning electron microscopy (SEM) to study the high spatial resolution (surface morphology) of the catalyst, XRD fortified thru Kά and Cu radiation source, enhanced at 20 mA and 40 kV, to institute the angular scanning electron implemented in the range of 10^o^ <2θ <80^o^ at speed of 2.5 ^o^C min^−1^ and to confirm the elemental composition of the sample and the quantitative structure of the sample. FTIR was used to check the presence of functional group and validate the presence of characteristic absorption bands of major elements present within the crystals powder structures. BET isothermal adsorption and Hammett indicator was used to establish the pore volume, the surface area, the basic density site, and the total basic density.

The method used for oil blend/mix BTO:WUVO in volumetric ratios as; 10:90 (BTO_10_)_,_ 20:80 (BTO_20_)_,_ 30:70 (BTO_30_)_,_ 40:60 (BTO_40_)_,_ 50:50 (BTO_50_)_,_ 60:40 (BTO_60_)_,_ 70:30 (BTO_70_)_,_ 80:20 (BTO_80_)_,_ and 90:10 (BTO_90_)_,_ respectively, to obtain low viscous oil, low acid value and accurate density required for transesterification of oil to biodiesel. The mixed oil in different ratios was properly was heated at 35 ^o^C on a hot plate for proper mixing owing to the uncertainty in the nature of fat. The viscosity, the acid value, and the specific gravity of the resulting mixed oil were examined.

Cocoa pod husk obtained from Cocoa processing factory in Ondo State, Nigeria. The pod husk was cleaned by washing with ionized water, and was decanted, kept overnight to allow proper draining. The drained-cleaned Cocoa pod was fermented in distilled water anaerobically (submerged) for 10 days. After which the fermented sample was separated from fermented water by decantation, dried in an oven at 120 ^o^C until a constant weight was achieved (bone dried). The dried fermented sample was milled and sieved into powder of 0.30 mm particle size before calcined at 750 ^o^C for 4 h in a furnace. The calcined sample (SFCTCPH) after calcination was left in the furnace for 24 h for proper cooling, and then placed in cleaned container for further analysis.

Production of biodiesel was carried out through the use of derived heterogeneous based CaO-catalyst synthesized from samples. The reaction process took placed in a 1000 ml reactor with three-necked, 200 ml of the oil mixed was first heated at 100 ^o^C for 60 min using a hot plate equipped with a magnetic stirrer. 2.5 (wt.%) of CaO catalyst was measured in a 250 ml dried-cleaned flask, and 50 ml of ethanol was measured and added to the ethanol flask to achieved EtOH/OMR of 1:4. The mix was placed on a shaker for 15 min, and then added to the heated oil in the reactor. The resulting mixed indicated two separated layers which contained the ethanol-catalyst layer and the oil layer. The reaction was carried out at 70 ^o^C for 65 min to reach completion.

At the end of reaction process, the non-soluble catalyst was removed by decanting, and the resultant product (ethanol-based-diesel) was distinguished through density separation. The biodiesel (fatty acid ethyl ester: FAEE) along with leached catalyst was separated by washing with warm mixture of 1.0 g sodium carbonate and 20 ml methanol. The washed mixed was separated by filtration, and the filtrate-diesel was washed with ionized water, and separated via gravity settling. The water wet-diesel was dried over anhydrous sodium sulphate (Na_2_SO_4_), and was separated by liquid–solid separation (decantation) to obtain the pure FAEE as liquid. The solid residual catalyst purified and reused. The step by step reaction process was conducted based on number of experimental runs generated by design expert.

The experimental data were used for the process optimization analysis of the FAEE production. The response variable was the FAEE yield, the input variables were the factors at five levels evaluated by mean of fit summary. The model was second-order, and the effects of variable significant and preferred terms were appraised by model effects. The ANOVA analysis (Analysis of Variance) was adapted to elucidate the data while diagnostic was used to estimate the fit of the model, and model transformation, the graphical plots were used to interpret and evaluates the model. The p-value called the probability value, the f-value called the factor value, the df known as degree of freedom, and the VIF called the variance inflation factor, were used for model significance. The regression parameters such as the coefficient of determination: $R^{2},$ the predicted coefficient of determination$:\;R_{pred.}^{2}$, the adjusted coefficient of determination: $R_{adj.}^{2},\;$and the adequate precision: Adeq. Prec., respectively, were used to check the model aptness.

Three-dimensional plots was used as a geometric setting to express the relationship between three variables while the second-order differential equation that further elucidates the connection between FAEE yield and the four factors is expressed arithmetically in Eq. (2).

(2) $$FAEE\;\left(\%\;wt.\right)=\varphi_{0}+\sum_{i=1}^{k}\varphi_{i}X_{i}+\sum_{i=1}^{k}\varphi_{ii}X_{i}^{2}+\sum_{i<j}^{k}\varphi_{ij}X_{i}X_{j}+R$$

Where FAEE is the yield in percentage, $\varphi_{0}$ is the cut off, $\varphi_{i}$ is the coefficient of linear variables, $\varphi_{ii}$ is the coefficient of interactive variables, $\varphi_{ij}$ is the coefficient of quadratic terms, $X_{i},\;X_{j}$ are the variables and R is the differential error.

The recovered catalyst was examined for its effectiveness by carried out the reusability tests. Catalyst purification of the recovered catalyst was carried out by the method used [11] with few modifications: the recovered catalyst was washed with an alcohol to eliminate the contaminant adhere at the catalyst interface as a data of transesterification processes. The catalyst purified with alcohol was centrifuged at 3500 rpm using an inbuilt heating vacuum centrifuge, and separated by decantation. The wet catalyst was dried in oven at 80 ^o^C for 60 min so as to make free of the alcohol before cooled temperature of 27 ^o^C and then reused.

Properties of the FAEE such as density, viscosity, moisture content, mean molecular mass, acid, saponification, iodine, peroxide, higher heating value, cetane number, API gravity, and diesel index were determined so as to determine its aptness as a substitute for conventional fuel used in diesel engine. These qualities were compared with [2] and [3] recommended standard.

The properties of mix oil ratio are displayed in Table 1, while Table 2 is the experimental design of four variables five level experimental design by response surface methodology. Table 3a displayed the experimental yield and the predicted value by the hybrid design. Table 3b reflected the test of significance by ANOVA, the probability values and the coefficient of determinations. Fig 1, displayed the three-dimensional contour of response on the variable factors (X_1_, X_2_, X_3_ and X_4_). The image of the SEM image and FTIR analysis of the calcined cocoa husk powder are displayed in Fig. 2(a-b). Table 4 showed the data of XRD and the BET analysis indicating the surface area, porous volume, basicity, and percentage compositions of the calcined catalyst. Reusability catalytic test strength data are displayed in Fig.4. Table 5 indicated the qualities of FAEE produced as compared with recommended biodiesel standard, while Table 6 compared the data of this study with the earlier reported work.

**Table 6:** Comparing this study with reported literature data.

Blended Oil	Blending ratio (vol/vol)	Catalysts	Calcination temperature and duration	% CaO/KOH conversion	Catalyst analysis	% Biodiesel yield	References
Waste cooking oil	-	Ripe and unripe Plantain peels	500°C for 4 h	KOH = 47.67%	XRD, SEM, BET, and FTIR	97.96	[6]
*Jatropha curcus+Heavea brasiliness+ Elais guineensis oils*	33:33:34	*Chiken foot, Cat fish bones, and mixed*	1000 ^o^C for 3 h	CaO = 99.84%	SEM, EDX-ray, FTIR and BET	97.25	[7]
*Calophyllum inophyllum*-waste cooking oil	50:50	Donax deltoids shells	105°C for 24 h	CaO = 70.87%	XRD, SEM, BET, and FTIR	96.50	[8]
Waste cooking oil (WCO)	-	Ca(NO_3_).4H_2_O	900°C for 3 h	CaO=99.92%	XRD, SEM, BET, and FTIR	99.19%	[9]
Waste + pure vegetable oil	-	Banana peel	700 ^o^C for 4 h	KOH		94 to 97%	[10]
Beef Tallow blend + Waste used vegetable oil	60:40	*Theobroma cacao* pod husks		CaO	XRD, SEM, BET, and FTIR		**THIS STUDY**
		Submerged fermented calcined	750 ^o^C for 4 h	87.65%		99.64%	

## Ethics Statement

This work does not involve the use animal or human subject.

## CRediT Author Statement

**Adepoju T.F.:** Conceptualization, Methodology, Software, Validation, Formal Analysis; **Ukpong A.A.:** Investigation, Resources, Data Curation, Writing – original draft, Supervision; **Balogun T.A.:** Validation, Formal Analysis, Investigation, Resources, Data curation; **Emberru E.R.:** Formal Analysis, Investigation, Resources, Data Curation, Provide Financial Support, Methodology, Software.

## Declaration of Competing Interest

The authors declare that they have no competing financial interests or personal relationships which have, or could be perceived to have, influenced the work reported in this article.

Financial institution. This research work receives no financial support from either Institution or government organization.

## Data Availability

Supfile (Reference data) (Github).

## Appendix Supplementary materials

Image, application 1
